# Editorial: Motor Skills and Their Foundational Role for Perceptual, Social, and Cognitive Development

**DOI:** 10.3389/fpsyg.2017.00301

**Published:** 2017-03-06

**Authors:** Klaus Libertus, Petra Hauf

**Affiliations:** ^1^Department of Psychology and Learning Research and Development Center, University of PittsburghPittsburgh, PA, USA; ^2^Department of Psychology, St. Francis Xavier UniversityAntigonish, NS, Canada

**Keywords:** motor development, developmental cascades, cognition, perception, social behavior, language development, autism spectrum disorders, developmental trajectories

Learning occurs through dynamic interactions and exchanges with the physical environment and the social world. In recent years, increasing attention has been paid to the contributions of a healthy and well-functioning motor system for children's learning. Consequently, it seems evident that motor development is critical for children's understanding of the physical and social world. The current Research Topic examines this timely question and provides a comprehensive overview of the current state of the field on the role of motor skills during development.

A total of 27 articles, covering applied and theoretical perspectives, are included in this Research Topic and examine the cascading influences of motor skills on perceptual, social, and cognitive processes. The contributions include both general overviews by review articles, presentation of novel findings in empirical articles, and opinion pieces that discuss future directions. Broadly speaking, the Research Topic covers three areas: First, articles that examine and describe the relation between motor skills and cognitive, perceptual, or social skills. Second, articles that discuss practical applications and implications by examining the role played by motor skills in developmental disorders. And third, articles that explore the direct effects of motor experiences on development across domains using training and enrichment paradigms. We will introduce and discuss each of these three areas in the following.

## How do motor skills influence cognitive, social, and perceptual development?

Previous research has identified close connections between motor experiences and development in other domains. As part of this research topic, Michel et al. review the literature on the development of infants' hand preference and find that those infants with consistent hand preferences early in development also show advanced cognitive development. This suggests that infants who show an early and stable hand preference may follow a different developmental pathway than infants who develop a hand preference later and provides evidence for the impact of motor experiences on cognitive development. However, this phenomenon is not limited to the development of handedness. For example, Libertus and Violi report that 3- to 5-month-old infants with faster rates of learning to sit independently show larger receptive vocabularies at both 10 and 14 months of age. Similarly, Ross-Sheehy et al. examined the relation between sitting and figure-ground assignment in 6.5-month-old infants and find that only infants who can sit independently use symmetry as a cue for figure-ground assignment. Together, these results indicate that the development of posture skills may alter infant's visual perception and language learning. Additional support for the importance of posture during learning comes from a study including both infants and robot models showing that posture can affect the mapping of words to objects (Morse et al., 2015). Walking is another motor milestone that has been shown to initiate developmental cascades affecting infants' social interactions (Karasik et al., 2014) and language development (Walle and Campos, 2014). In this collection, Walle examined the effects of walking acquisition on social development *and* parent's perception of their child by longitudinally following infants from 10.5 to 13 months of age. Results show an increase in infants' joint engagement (both initiation and following) with increasing walking experiences, and an increase in parent's perception of their child as an individual following walking onset. This provides evidence for the cascading effects that motor experiences have on infant's own behavior and the behavior of parents and others interacting with the child. Similarly, Karasik et al. examined how locomotor experience (either crawling or walking) affects infants' extraction and use of information to guide their future actions. Their results show that infants with more locomotor experiences were more likely to use social information to guide their actions than novice walkers.

Together, the studies summarized above provide strong evidence for a relation between motor experience and cognitive, social, or perceptual development in infancy. However, other contributions in this Research Topic show such a relation becomes less clear in older children. For example, Nakagawa et al. examined how temperament is related to both motor coordination and sensory processing in 3-year-old children. Their results suggest that effortful control (i.e., temperament) affects *both* motor and sensory domains. Thus, it is possible that motor experiences and perceptual development appear related because both are affected by a child's temperament. Similarly, De Jonge-Hoekstra et al. conducted real-time observations between gestures and speech in 4 to 7-year-olds and report changes in gesture-language synchrony over time. This suggests that the influences of motor experiences on other domains may change as children grow older. In fact, Oudgenoeg-Paz et al. examined the relation between walking onset and subsequent linguistic skills at 3.5 years of age and found no relation between age of walking onset and language at 43 months of age. This contrasts with previous studies reporting a positive relation between these skills in infancy (e.g., He et al., 2015) and suggests that the relation between motor and language development may *diminish* over time. Supporting this view, Kenny et al. examined the relation between social cognition (i.e., theory of mind) and motor skills in primary school aged children and reported *no relation* between motor and social skills. This argues that social and motor processes may be independent in school aged children. Similarly, Hagmann-von Arx et al. quantified gait characteristics in 6.7–13.2 year-old children and found that gait alterations were not related to children's cognition or psychosocial functioning. Thus, despite the growing evidence for associations between motor and cognitive, social, or perceptual development during the first 2–3 years of life, this relation seems absent or at least masked once children enter Kindergarten.

However, there is also evidence that connections between motor experiences and cognitive development may exist beyond infancy but are limited to specific domains of cognition. Specifically, two studies in this Research Topic provide evidence for a relation between motor development and mathematics in Kindergarten and primary school aged children. First, Frick and Möhring report that children's ability to balance on one leg at age 6 years predicts their proportional reasoning skills assessed 1 year later. And second, Pitchford et al. report that fine motor skills (i.e., Fine Motor Precision and Fine Motor Integration) were a significant predictor of math abilities in 4- to 6-year-old primary school students. These studies suggest that motor skills can be predictive of cognitive skills beyond infancy—at least in the domain of mathematical cognition.

Together, the studies reported in this collection suggest an interesting shift in the relation between motor skills and other developmental domains over time. Specifically, motor skills seem highly related to other developmental domains during the first 3 years of life, but this relation seems to weaken or disappear as children grow older—with the one notable exception being a positive relation between motor and math skills that persists until at least age 6.

## Are motor skills impaired in developmental disorders?

If motor experiences are indeed important for cognitive, social, or perceptual development, it should be expected that delays in motor skills are associated with impaired cognitive, social, or perceptual development as well. Three review articles present evidence for this position. First, Einspieler et al. review findings showing that general movements as assessed using The Prechtl General Movement Assessment at 2, 3, or 5 months of age are predictive of subsequent cognitive development in children born preterm. This shows that the infant's motor repertoire during the first months of life is predictive of cognitive outcomes. Second, Leonard reviews evidence for impaired perceptual, social, and cognitive skills in children with Developmental Coordination Disorder (DCD). And third, Mancini et al. review evidence supporting the Elaborated Environmental Stress Hypothesis which suggests that poor motor skills predispose children for subsequent internalizing problems. These reviews strongly support that poor motor skills may be predictive of impaired cognitive, social, or perceptual development.

Further support for the notion that poor performance in basic motor skills may be predictive of developmental delays in other domains comes from research with infants later diagnosed with Autism Spectrum Disorder (ASD). In the current collection, Landa et al. examined the presence of motor delays in ASD and report that 6- and 14-month-old infants at high familial risk for ASD (HR infants) show reduced anticipatory motor responses during a social interaction involving an object. Similarly, St. John et al. report poor executive functioning (EF) in HR infants at 24 months of age. Critically, poor motor skills at both 12 and 24 months were associated with poor EF performance in HR infants. Motor skills in ASD were also examined by Focaroli et al. who recorded movement kinematics during object exploration in HR children at 18, 24, and 36 months. In their study, HR children showed slower reaching movements compared to low-risk children, providing further evidence for delayed fine motor skills in ASD. And finally, Mancini et al. provide empirical evidence for the Elaborated Environmental Stress Hypothesis by showing that poor motor skills can indirectly increase depression in adolescents by influencing perceived family support. Together, these studies show that motor delays and abnormalities are common in children with developmental disorders and that cognitive, social, or perceptual delays are often preceded and potentially exasperated by motor deficits.

## What experiences affect children's motor development?

The papers presented in this Research Topic show that motor skills are important for a child's healthy development across domains and that early motor delays may be predictive or elevated risk for developmental disorders or mental health problems later in life. These observations raise the question: Can we improve a child's motor skills and should intervention efforts include this domain? The short answer to both questions is yes. Indeed, several studies included in this Research Topic show that training motor skills is both feasible and effective. Wiesen et al. provided 3-month-old infants with scaffolded reaching experiences using ‘sticky mittens’ (Needham et al., 2002; Libertus and Landa, 2014) and examined training effects immediately after training and 2 months later. Their results show that trained infants showed improved object engagement and exploration, both immediately after training and after a 2-month delay (without further training in the meantime). This confirms previous results and shows that early motor training can be beneficial for typical developing infants (e.g., Libertus et al., 2016). Ryalls et al. report on the effects of sitting training in children with moderate or severe cerebral palsy (CP). Their results show that sitting skills could be improved in children with CP (ages 18 months to 6 years) using a perceptual motor intervention. More importantly, improvements in children's sitting skills were also associated with improvements in functional play skills in children 3 years of age or older—skills that may support future learning and development. And finally, Pesce et al. provided 5- to 10-year-old children with enriched physical education (PE) and examined the impact of this experience on their cognitive abilities. Results show that children who received the enriched PE improved their motor coordination skills, and in those children who received enriched PE and showed higher levels of spontaneous outdoor play an improvement in inhibition skills was observed as well. These findings indicate that motor enrichment can have positive influences on children's cognitive development, but only if the children are already physically active on their own. Consequently, motor activity and engagement should be encouraged in all children and may increase the effectiveness of instruction or intervention procedures.

## Open questions

The findings summarized in this Research Topic address several timely questions on the role of motor experiences during development and increase our understanding of the developmental process and its dynamic connections across domains that are typically studied in isolation. However, several questions remain unanswered. For example, the mechanism behind the observed relations between the motor and other domains remains poorly understood. Studies on the neural basis of such relations are needed to address this question. In this collection, Gonzalez et al. summarize findings that examine the effect of motor experiences on the brain by using electroencephalography (EEG) measures such as power, coherence, and my desynchronization. Unfortunately, findings to date are not conclusive and the authors note that more longitudinal research is needed to understand the neural mechanism underlying developmental cascades initiated by motor experiences.

Further, the various factors that influence motor development itself remain largely unknown as well. For example, what is the role of mother's behavior and health for a child's motor development? Piallini et al. show that even sub-clinical symptoms of psychopathology in the mother can affect infant's motor development during the first year of life. These findings highlight the importance of the mother-child dyad and their interactions for a child's development over time.

While the findings presented in this Research Topic indicate that good motor skills are associated with positive developmental outcomes in other domains, it remains unclear whether motor skills are directly related to cognitive, social, and perceptual development, or whether motor skills and the other domains are both influences by a third factor—such as the mother's health. Given the results of the training studies reported here and elsewhere, it seems likely that motor experiences have at least some direct influence on development in other domains. However, future research is needed to investigate this hypothesis. We hope that the collection presented in this Research Topic will encourage such research and stimulate a broader discussion on the importance of motor experiences during childhood in our daycares and school settings.

## Author contributions

KL wrote the first draft of this editorial and PH edited consecutive versions. Both authors have agreed on the final version. The overall research topic has been conceptualized equally by KL and PH.

## Funding

KL has been supported by a Slifka/Ritvo Innovation in Autism Research Award.

### Conflict of interest statement

The authors declare that the research was conducted in the absence of any commercial or financial relationships that could be construed as a potential conflict of interest.
